# An Analysis of the Risk Factors for the Development of Parastomal Hernia: A Single Institutional Experience

**DOI:** 10.7759/cureus.21470

**Published:** 2022-01-21

**Authors:** Faiza H Soomro, Sufyan Azam, Sritharan Ganeshmoorthy, Peter Waterland

**Affiliations:** 1 General Surgery, The Dudley Group National Health Service Foundation Trust, Dudley, GBR; 2 Surgery, Queen Elizabeth Hospital Birmingham, Birmingham, GBR; 3 Colorectal Surgery, The Dudley Group National Health Service Foundation Trust, Dudley, GBR; 4 General Surgery, Russells Hall Hospital, Dudley, GBR

**Keywords:** malignancy, trans-rectus, junctional, risk factors, parastomal hernia

## Abstract

Objectives: To study the frequency of risk factors affecting the development of parastomal hernias in patients undergoing stoma formation.

Study Design: A retrospective descriptive cross-sectional study.

Duration of Study: This study was conducted at the Department of General Surgery between January 2017 to December 2020.

Methodology: A total of 163 patients aged between 20 and 100 years and who required a stoma formation were included in the study. The patients with incomplete data and those lacking post-operative imaging were excluded. According to this selection criteria, 80 patients were excluded. The data was collected for all patients from the hospital database. This included patient’s demographic information, co-morbidities, pre-surgery patient characteristics, an indication of stoma formation, the location of stoma exit, type of surgery, associated comorbidities, subcutaneous fat thickness, and type of stoma formed. Data were analyzed using IBM Corp. Released 2019. IBM SPSS Statistics for Windows, Version 26.0. Armonk, NY: IBM Corp.

Results: The mean age was 68.46 ± 16.50 years, with males in the majority: 48 (57.8%). Most of the patients, 53 (63.8%), had malignant disease. Post-stoma formation, a total of 38 (45.9%) patients developed parastomal hernias, mostly involving the sigmoid colon (n=62, 74.7%). However, there was a statistically significant relationship between paroxysmal sympathetic hyperactivity (PSH) incidence with non-trans-rectus stomas (trans-oblique n=07, junctional n=28) (OR 3.04, CI 1.23-7.5, p=0.014). Furthermore, malignancy was also not an independent predictor of PSH (OR 0.408, CI 0.15-1.2, p=0.056). All other risk factors included in this study were nonsignificant.

Conclusion: Our study shows that the incidence of parastomal hernias is rising with a high rate demonstrated in our patients. There was no statistically significant association between patient-related preoperative and operative factors with increased risk of parastomal hernias in our population except for a non-trans-rectus stoma, which was identified as an independent risk factor for parastomal hernias. Based on our findings, we would recommend a trans-rectus stoma over all other stoma sites. However, a much larger study is needed to validate this finding further.

## Introduction

Stoma formation is among the most commonly performed surgical procedures to redirect gut contents for various reasons [1]. Parastomal hernia is characterized as the protrusion of abdominal contents through the abdominal wall defect in the locality of the stoma [2]. Parastomal hernias complicate stomas at an incidence of as high as 0% to 48% for end colostomies, 0% to 30.8 % for loop colostomies, and 1.8% to 28.3% for end ileostomies [3]. There are high chances that parastomal hernias may remain asymptomatic. However, there are also chances that they may become obstructed or strangulated creating life and death scenarios [4]. Sohn et al. reported that 37.8% of their patient population who had stomas after surgery developed parastomal hernias; they identified female gender, advancing age, a BMI > 25 kg/m2, and hypertension as independent risk factors for the development of a parastomal hernia [5]. In contrast, Pennings et al. reported chronic obstructive pulmonary disease (COPD), long duration of surgery, and large diameter of stoma as independent risk factors for parastomal hernia development [6].

It is reasonable to recognize the risk factors for parastomal hernias earlier and reduce the morbidity and mortality associated with this complication in colorectal surgeries. Hence, we conducted a retrospective study to investigate the frequency of occurrence of parastomal hernias in our center along with the possible risk factors in our community.

## Materials and methods

**Figure 1:**
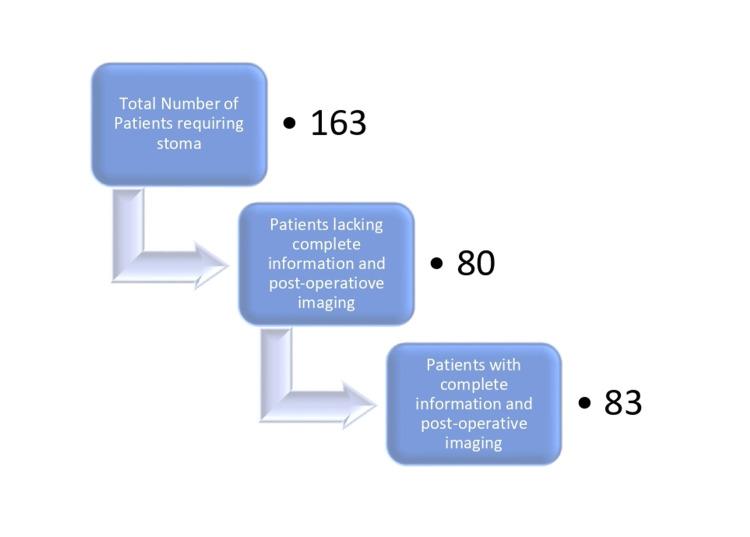
Inclusion and Exclusion Criteria

This was a retrospective, descriptive cross-sectional study conducted from January 2017 to December 2020, which amounted to four years in the Department of General Surgery, Russells Hall Hospital, Dudley. All patients between 20 and 100 years with at least one year of completed follow-up that required a stoma formation as a part of the first operation were included in the study. A total of 163 surgeries for the construction of intestinal stomas were performed during the study period. Out of 163 patients, 29 patients didn’t have complete information, and 51 patients had no post-operative imaging, so these were excluded. The remainder of 83 patients with comprehensive database and post-operative imaging were included in the study (Figure 1).

The data was collected for all patients from our hospital database. The patient’s demographics included age, sex, and American Society of Anesthesiologists (ASA) criteria. Pre-surgery features included co-morbidities, the indication of surgery, the etiology (whether benign disease or malignant), and the confidential enquiry into perioperative deaths (CEPOD) level of intervention. Operative characteristics included the location of stoma exit, type of surgery, subcutaneous fat thickness, muscle thickness, and type of stoma formed. The primary outcome measure was to see if the site of stoma formation has any impact on parastomal hernias. CT scan was used as an adjunct to establish, if any, the relation between the fat thickness and the muscle thickness measured at the site of parastomal hernia. 

Data were analyzed using IBM Corp. Released 2019. IBM SPSS Statistics for Windows, Version 26.0. Armonk, NY: IBM Corp. Mean and SD was calculated for quantitative variables, while qualitative variables were recorded in frequency and percentage. An independent sample t-test was applied for comparison between the groups, and the signiﬁcance level was determined to be < 0.05. Analysis was completed using IBM Corp. Released 2013. IBM SPSS Statistics for Windows, Version 22.0. Armonk, NY: IBM Corp.

## Results

We studied 83 patients, with a mean age of 68.46 ± 16.50 years. Males accounted for 57.8% (n=48) of these patients. The majority of the patients were white: 96.4% (n=80), 48.2% (n=40) suffered from diabetes mellitus. A total of 22 (26.5%) were ASA class I, while class II and III were 36 (43.3%) and 25 (30.2%), respectively.

The majority of cases were performed electively: 57 (68.6%). The most frequently performed procedure was Hartmann’s procedure: 34 (41%), followed by abdominoperineal resection, which accounted for 32 (38.6%) cases. Colon was brought up to form a stoma in 61 (73.5%). In 49 (59.1%), end stomas were formed, while the rest were loop ostomies. A majority of the stomas were trans-rectus 48 (57.7%). A total of 38 (45.9%) patients developed parastomal hernias, most of which involved the sigmoid colon (n=62, 74.7%).

Increasing age, gender, diabetes mellitus, etiology, CEPOD level, operative time, or type of stoma did not correlate with increased risk of parastomal hernia formation, as shown in Tables 1, 2. However, there was a statistically significant relationship between PSH incidence with non-trans-rectus stomas (trans-oblique n=07, junctional n=28) (OR 3.04, CI 1.23-7.5, p=0.014) shown in Table 1.

**Table 1:** Comparison of risk factors of Parastomal Hernia using (categorical data) PSH: parastomal hernia, ASA: American Society of Anesthesiologists

	Patients with PSH	Patients without PSH	Confidence Interval	Odd’s Ratio	P value
Gender					
Male	22	26	0.60-1.44		0.11
Female	16	19	0.601-1.65		
Co-Morbids					
Diabetes Mellitus	19	21	0.48-2.71	1.14	0.762
ASA					
ASA I	09	13			0.865
ASA II	17	19			
ASA III	12	13			
Type of Surgery					
Emergency	13	13	0.30-1.98	0.78	0.603
Elective	25	32			
Stoma Location					
Colonic	30	31	0.621-4.61	1.69	0.301
Small Bowel	8	14			
Stoma Exit					
Trans-rectus	16	31	1.23-7.5	3.04	0.014
Non-trans-rectus	22	14			
Stoma Type					
End Stoma	26	23	0.84-5.09	2.07	0.11
Loop Stoma	12	22			
Etiology of Stoma					
Malignant	20	33	0.15-1.-2	0.408	0.056
Non-malignant	18	12			

**Table 2:** Analysis of risk factors for Parastomal Hernia (numerical data) PSH: parastomal hernia

	Mean	Standard Deviation	Confidence Interval	P Value
Age				
Patients with PSH Hernia	70.32	16.051	3.81-10.66	0.349
Patients without PSH	66.89	16.88		
Total Operation Time				
Patients with PSH Hernia	58.82	13.03	7.41-4.37	0.610
Patients without PSH	58.34	13.09		
Fat Thickness				
Patients with PSH Hernia	28.03	15.06	0.55-11.63	0.074
Patients without PSH	22.49	12.44		
Rectus Thickness				
Patients with PSH Hernia	8.33	2.55	0.39-1.89	0.198
Patients without PSH	7.58	2.66		

We found no statistically significant association regarding fat thickness and rectus muscle thickness calculated from the CT scan (P=0.074; P=0.198), respectively, which has been shown in Table 2.

## Discussion

The incidence of parastomal hernias has been on the rise in recent decades, not only due to increased surgeries resulting in the formation of stomas but also due to the frequent employment of imaging, such as computed tomography, for diagnosis of even small-sized hernias [7]. Our study saw an incidence rate of 45.8%, which was much higher than other studies because we used CT for diagnosis. In contrast, other studies established diagnoses based on clinical findings [8,9]. CT has been shown to have a high sensitivity and specificity in diagnosing abdominal wall hernias, with little inter-observer variability [10]. Longer follow-up times have also been associated with an increased incidence of parastomal hernias, up to five years, after which the incidence tends to drop dramatically [7,11]. Surgical management of parastomal hernias has been associated with higher recurrence rates globally, as per the available literature [12]. These numbers have prompted the surgeons to go to primary measures that can be done to prevent it from happening in the first place. There is growing evidence that the placement of mesh at the time of stoma creation decreases the incidence of stoma creation [13,14].

Our study showed a positive relationship between the development of parastomal hernias and the location of the exit of the stoma, with a lower risk of occurrence if it was trans-rectus compared to trans-oblique or trans-oblique junctional stomas. Previous studies have shown that citing stoma outside the rectus muscle is associated with an increased risk of developing hernias [15]. While other studies have shown positive associations between preoperative patient-related factors and a higher risk of parastomal hernias [16], we could not find any significant association among all the other outcomes of interest in our study population. Other factors need further consideration in terms of parastomal hernias, such as peristomal skin disorders; although studies show that parastomal hernias cause leakage and increase the risk of peristomal skin disorders, the opposite might not be accurate [17]. Furthermore, we did not compare the risk of parastomal hernias between open and laparoscopic approaches. There is growing evidence that the laparoscopic approach might be associated with an increased risk of parastomal hernias, particularly for trans-rectus stomas, this can be attributed to the fact that patient sometimes is not entirely supine and the remaining pneumoperitoneum may make it difficult for the stoma to pass through the middle of the rectus, thus increasing the risk of PSH. [18]. This, however, needs to be further validated. 

It is highly likely that due to a limited number of patients and retrospective study design, we could not successfully identify any causal associations. However, it is essential to be aware of the findings of this study. We recommend a prospective study with a larger cohort of patients to ensure extra precautions can be taken to identify and address the patient-related risk factors and improve outcomes for patients who already have to bear the burden of reduced quality of life secondary to stoma formation.

## Conclusions

Our study shows that the incidence of parastomal hernias is on a rise, with a high rate shown in our patients as well. There was no statistically significant association found between patient-related preoperative as well as operative factors with increased risk of parastomal hernias in our population except for a non-trans-rectus stoma, which was identified as an independent risk factor for parastomal hernias. Based on our findings, we would recommend a trans-rectus stoma over all other stoma sites; however, a much larger study is needed to further validate this finding.
